# A nosographic and etiopathogenetic framework for subchondral bone marrow lesions in the knee: A narrative review

**DOI:** 10.1002/jeo2.70071

**Published:** 2025-01-16

**Authors:** Luca Andriolo, Alessandro Sangiorgio, Anthony Galea, Eran Linder‐Ganz, Patrick Orth, Nogah Shabshin, Takuaki Yamamoto, Giuseppe Filardo

**Affiliations:** ^1^ Clinica Ortopedica e Traumatologica 2, IRCCS Istituto Ortopedico Rizzoli Bologna Italy; ^2^ Service of Orthopaedics and Traumatology, Department of Surgery EOC Lugano Switzerland; ^3^ IHM Clinics Toronto Ontario Canada; ^4^ Active Implants Netanya Israel; ^5^ Department of Orthopaedic Surgery Saarland University Medical Center Homburg/Saar Germany; ^6^ Department of Radiology Carmel Medical Center Haifa Israel; ^7^ Department of Orthopedic Surgery Fukuoka University Faculty of Medicine Fukuoka Japan; ^8^ Faculty of Biomedical Sciences, Università della Svizzera Italiana Lugano Switzerland; ^9^ Applied and Translational Research (ATR) Center, IRCCS Istituto Ortopedico Rizzoli Bologna Italy

**Keywords:** bone marrow lesions, knee, osteoarthritis, subchondral bone

## Abstract

**Purpose:**

Subchondral bone marrow lesions (BMLs) are present in a wide range of pathologies with different prognoses. Thus, a careful diagnosis is mandatory to address them with the proper treatment. The aim of this review was to examine BMLs aetiology and their relationship with biomechanical and biological factors, to identify BMLs and help clinicians to properly recognize and treat each of these common alterations.

**Discussion:**

Each pathological pattern is determined by different aetiologic factors, which may act alone or synergically in determining the BML. These factors include major or minor trauma, bone tissue alterations, altered joint load distribution, coagulopathies, and hormonal alterations. This narrative review encompasses these patterns and factors providing a nosographic and etiopathogenetic framework for subchondral BMLs in the knee.

**Conclusion:**

While the field is still heterogeneous in the definition of the nosographic framework of BMLs, there is a trend with the convergence towards a common terminology, which could help to shed more light on the complex and varied field of BMLs. Future studies should focus on better understanding the etiopathogenetic mechanisms, which can concur with the development of BML from one side, and, on the other hand, may represent targets for future treatments to address BMLs and preserve or restore the osteochondral unit.

**Level of Evidence:**

Expert Opinion, Level V.

**Abbreviations**
ACL: anterior cruciate ligament
AVN: avascular necrosis
BML: bone marrow lesion
CRPS: complex regional pain syndrome
GH: human growth hormone
IGF‐1: insulin‐like growth factor 1
IL‐1: interleukin 1
MMP: matrix metalloproteinase
MRI: magnetic resonance imaging
OA: osteoarthritis
PTH: parathyroid hormone
RMO: regional migratory osteoporosis
SIFK: subchondral insufficiency fractures of the knee
SONK: spontaneous osteonecrosis of the knee
STIR: short tau inversion recovery
T3 and T4: thyroid hormones
TLD: tibia‐lateral‐articular distal lesion
TOP: transient osteoporosis
VEGF: vascular endothelial growth factor

## INTRODUCTION

Bone marrow lesions (BMLs) of the knee are a common magnetic resonance imaging (MRI) finding. BML is defined as an alteration of the bone marrow signal intensity, with high signal on fluid‐sensitive sequences (T2/proton density with fat suppression and short tau inversion recovery—STIR) with or without low T1WI signal [30]. These MRI alterations may correspond histologically to true oedema, but also to trabecular necrosis, cysts, fibrosis, and cartilage fragments. Therefore, instead of the commonly used term ‘bone marrow oedema’, the expressions ‘bone marrow oedema‐like signal’ or ‘BMLs’ are more appropriate [80].

Subchondral BMLs gained increasing attention among the scientific community, as they are present in a wide range of pathologies, including traumatic contusions and fractures, post‐cartilage surgery alterations, osteoarthritis (OA), transient BMLs syndromes, subchondral insufficiency fractures of the knee (SIFK), and spontaneous osteonecrosis of the knee (SONK) [40, 64]. Within these pathologies, different aetiologic factors may act alone or synergically in determining the BML. These factors include major or minor trauma, bone tissue alterations, altered joint load distribution, coagulopathies, and hormonal alterations. Since these pathological patterns present different prognoses, a careful diagnosis is mandatory to address them with the proper treatment. Together with the patient's clinical history and objective evaluation, MRI plays a fundamental role in guiding the diagnosis based on recognizable typical patterns even at early stages. In‐depth knowledge is fundamental to set up the most appropriate diagnosis and therapeutic strategies.

To serve this purpose, this narrative review aims at clarifying BMLs aetiology and the relationship with biomechanical and biological factors. In the next paragraphs, BML characteristics, incidence and pathogenesis will be examined, analysing traumatic lesions, non‐traumatic lesions, the effect of subchondral bone load distribution, coagulopathies, and hormonal factors on bone marrow lesion development, to help clinicians to properly recognize and treat these common alterations.

## TRAUMATIC LESIONS: PREVALENCE, EVOLUTION AND IMPACT OF BONE BRUISE

Trauma‐induced BMLs are associated with acute direct or indirect trauma (i.e., bone contusions) or with subacute progressive lesions as a result of overload (i.e., stress fractures and repetitive microtrauma occurring during physical activity). The incidence of BMLs is directly related to trauma mechanism and force, with a frequent association with knee ligament tears [74], although the presence of subchondral oedema‐like lesions was also described in up to 41% of asymptomatic basketball players subject to repetitive microtrauma [39].

The most common subchondral bone contusions are those seen after pivot shift injuries, often associated with anterior cruciate ligament (ACL) tears, even in the paediatric population [12, 45, 72, 74]. In fact, the large external forces responsible for ACL rupture cause a violent impact between tibial and femoral articular cartilage, which is transferred to bone, resulting in BMLs (also known as bone bruises) in 78% of ACL injuries [25]. In these cases, BMLs are mainly sited on the mid‐portion of the lateral femoral condyle and the posterior lateral tibial plateau: this pattern is the result of the typical valgus stress occurring in an ACL injury with the femur in external rotation relative to a fixed tibia, which explains why the lateral compartment is more involved than the medial one [13]. Other location‐specific patterns are described in case of hyperextension injuries [63], which may lead to ligament tears and cause subchondral contusions in the anterior tibia and femur, or in case of a spontaneously reduced lateral patellar dislocation in teenagers around the physeal closure. The latter is characterized by one or a combination of the following: a kissing impaction in the medial patellar facet or the median ridge, a medial patella traction lesion of the medial retinaculum, impaction contusion in the anterior lateral femoral condyle, and an osteochondral lesion in the lateral femoral condyle associated with a sequestered intra‐articular fragment [62].

Subchondral bone injuries following a single direct impact or resulting from repetitive microtrauma show peculiar histopathological features. The underlying bone is locally impacted, presenting microfractures of the subarticular spongiosa, with osteocyte necrosis and empty lacunae, haemorrhage and oedema [57]. These histological findings correspond to the MRI pattern. If the traumatic impact is more severe, a subchondral fracture may cause local depression and even collapse of the cartilage surface. Fractures affecting the osteochondral unit (either with a chondral or osteochondral fragment, or purely subchondral) may show accompanying BMLs. When, in the course of bone remodelling, the subchondral bone becomes stiffer, OA signs may appear. In this case, the cartilage overlying such areas is directly affected as well, with chondrocyte apoptosis and necrosis, chondrocyte proliferation, and loss of superficial proteoglycans [47].

The natural history of post‐traumatic bone contusions has been poorly investigated, especially in the long term. BML evolution is influenced by several factors. While BML associated with isolated medial collateral ligament tear may spontaneously heal in 2–4 months, it has been reported that BML in a complex knee injury with ACL tear has a slower resolution [43]. Moreover, BML in ACL lesions is predominantly present at 3 years of follow‐up when associated with a disruption or a depression of the normal contour of the femoral cortical surface, while lesions without cortical involvement tend to have spontaneous resolution in 95% of cases [21]. In addition, the location of the lesions may affect the evolution of BML. In fact, 67% of lateral femoral condyle bone bruises associated with ACL injuries tend to evolve into osteochondral damage [73]. Finally, post‐traumatic BMLs in the lateral femoral condyle in ACL‐injured knees showed a quicker resolution compared to lateral tibial BMLs (median 3 vs. 6 months) [26].

According to the few studies reported in the literature, there is no agreement about a correlation at short‐term follow‐up between BMLs, pain and functional status, even though it has been reported that BML may negatively affect pain, functional recovery, and return to previous sport level, especially in case of large BML, medial side distribution, and if the alteration is still detectable 3 months after injury [24, 28]. Similarly, a systematic review of 83 papers analysing bone bruises associated with ACL injuries reported that BMLs were detectable only in a minority of cases the first few months after trauma, but their presence and persistence were correlated to more severe joint damage that may lead to the degenerative progression of the entire joint. This may suggest possible effects on long‐term clinical outcomes [25], with a lower return to sport at mid‐term follow‐up after ACL reconstruction in joints presenting bone bruises at baseline MRI [30]. Still, it remains unclear if the initial joint injury and BML characteristics are directly correlated to long‐term functional outcomes and OA development.

## NON‐TRAUMATIC LESIONS: AN ETIOPATHOGENETIC FRAMEWORK AND THE PROBLEM OF TERMINOLOGY

The presence of BMLs represents a frequent finding also in joints without a history of trauma. There is actually a wide spectrum of different conditions that cause non‐traumatic oedematous signals involving an articular surface, almost always of a lower extremity, on MRI. The included conditions are transient osteoporosis (TOP), regional migratory osteoporosis (RMO), insufficiency fractures, complex regional pain syndrome (CRPS), also known as algodystrophy, osteonecrosis and avascular necrosis (AVN). There is confusion and sometimes overlap in the terminology and communications, different articles use different names for the same condition, thus limiting the development of systematic research in this field. However, the major classification of BMLs as reversible and irreversible has reached a consensus. Reversible BML includes TOP, RMO, and CRPS. Irreversible BML includes primary and secondary osteonecrosis. Primary osteonecrosis is a synonym for AVN, and an underlying condition is almost always present. Such conditions include steroid treatment, lymphoma treatment (that includes steroids), and bone marrow replacement or hypercoagulability. Secondary osteonecrosis is a complication of an insufficiency fracture, in which the subchondral fracture fragment undergoes necrosis. SIFK can be reversible, but can also progress to secondary irreversible osteonecrosis [46].

### Reversible versus irreversible conditions

On MRI, all these conditions present with subchondral BMLs, and the reversible conditions may look exactly the same. When trying to differentiate between reversible and irreversible conditions, many factors must be taken into account, such as patients’ age and gender, underlying diseases, concomitant medications, and prior trauma. Bone density measurements are still in debate and therefore do not help in reaching the diagnosis. Recently, local bone density measurements performed on two patients with transient bone marrow oedema of the knee demonstrated focal osteopenia [10]. TOP usually affects the femoral head of middle‐aged men and pregnant or peripartum women. The natural course of this entity shows spontaneous remission after 6–12 months [2]. If a similar episode has already happened before at a different joint, and even more if the patient is a middle‐aged man, the correct term to use would be RMO. When there is an underlying pain initiator, the patient may be suffering from CRPS, even if there are no specific characteristics for this condition detectable on routine MRI. A dedicated CRPS MRI protocol that compares the bilateral extremities can sometimes demonstrate diffuse skin thickening or muscle atrophy. When a subchondral fracture line or flattening of the articular surface is seen, especially in patients aged over 60, this is a SIFK. It is somewhat more challenging to define whether SIFK is reversible or not. If there is a band of subchondral low bone signal on all sequences, which is thicker than 4 mm, the lesion is irreversible. On the other hand, if there is no such band and the articular surface shape is preserved, the SIFK is reversible. At the end of the spectrum lies AVN, which has the typical appearance of the serpiginous double line sign [44]. For this condition, diagnosis is made with the presence of underlying disease or medications, and age does not matter. There is probably an overlap between the different syndromes, and further research may shed light on this group of conditions.

### A problem with terminology

This explains some confusion and the evolution of the BMLs' interpretation over time, especially when considering the concepts of SONK, AVN, and SIFK. The clinical condition called SONK was first reported in 1968 [1] and included the sudden onset of knee pain in elder patients, especially in the female sex, without a usual association with prior corticosteroid intake. The lesion was generally seen at scintigraphy or MRI in the medial femoral condyle, and in the initial phases after pain onset, the radiographs showed no obvious alterations. In the original paper [1], the concept of osteonecrosis/AVN was proposed mainly based on increased isotope uptake in one portion of the knee. In addition, needle biopsies performed on seven patients were interpreted as AVN based on the presence of some empty lacunae. However, more definitive features of AVN, such as creeping substitution and bone marrow necrosis, have been described. In spite of such histological uncertainty, this necrosis had been initially considered as a result of ischaemia, thus being called ‘avascular’.

**Figure 1:**
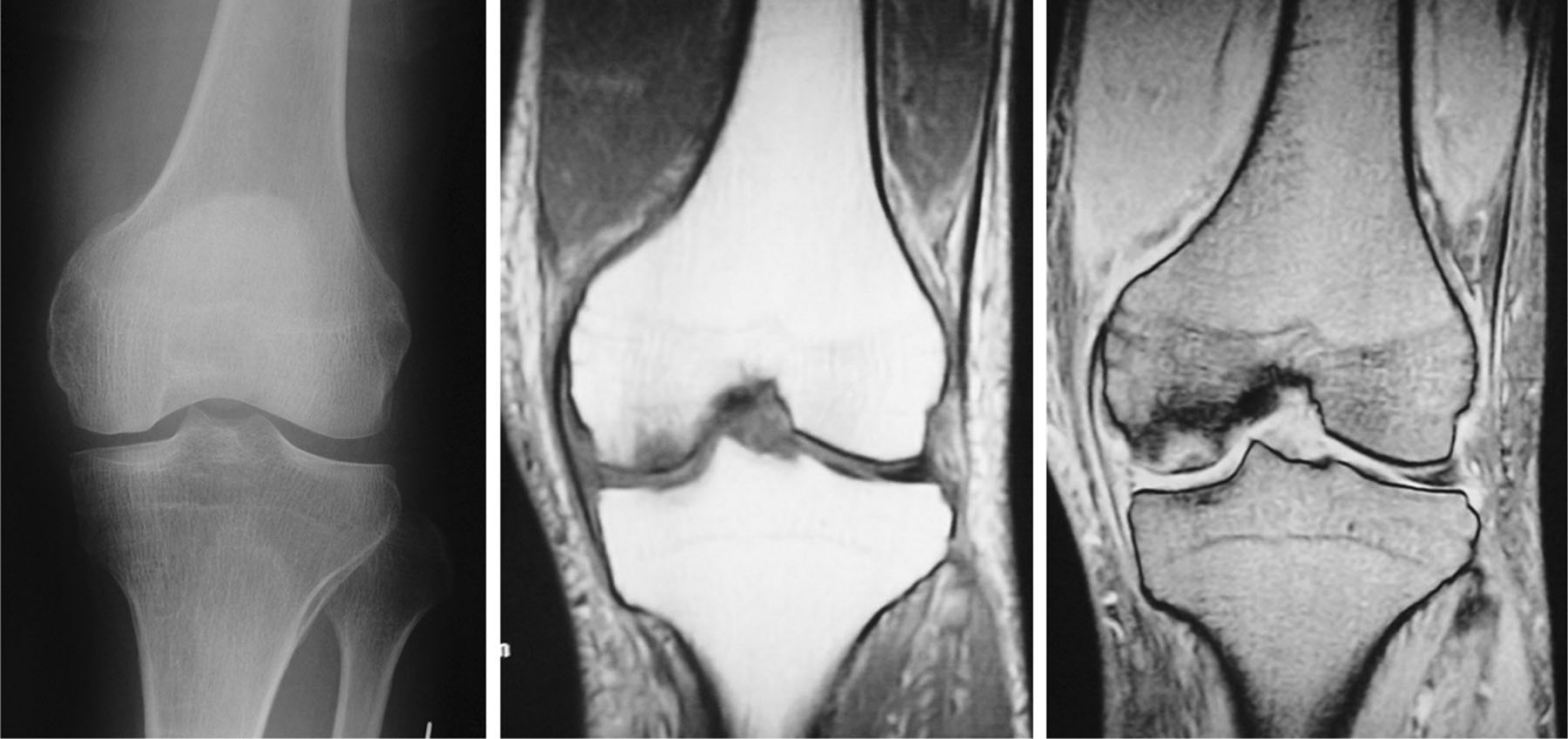
Subchondral insufficiency fracture on plain radiographs and magnetic resonance imaging of a 64‐year‐old male patient.

**Figure 2:**
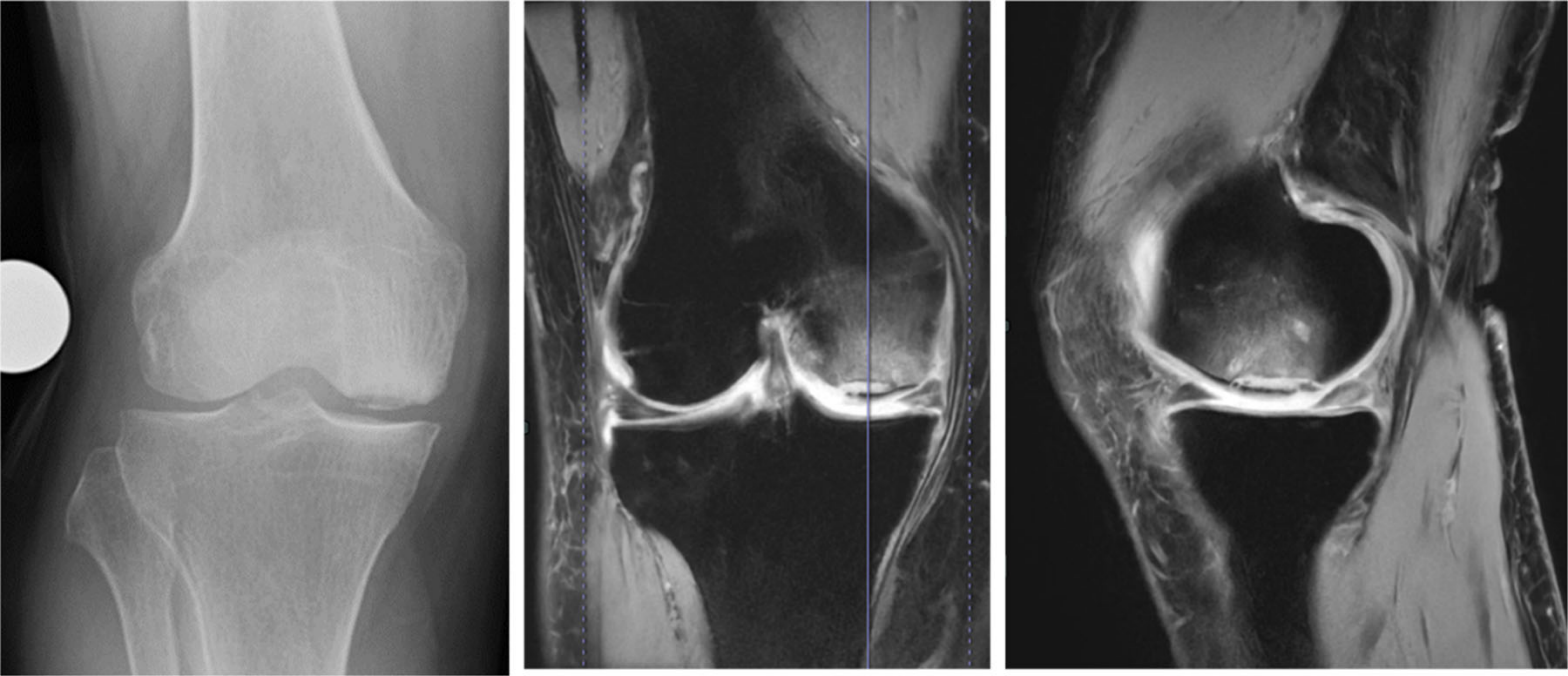
Subchondral insufficiency fracture on plain radiographs and magnetic resonance imaging of a 72‐year‐old female patient.

**Figure 3:**
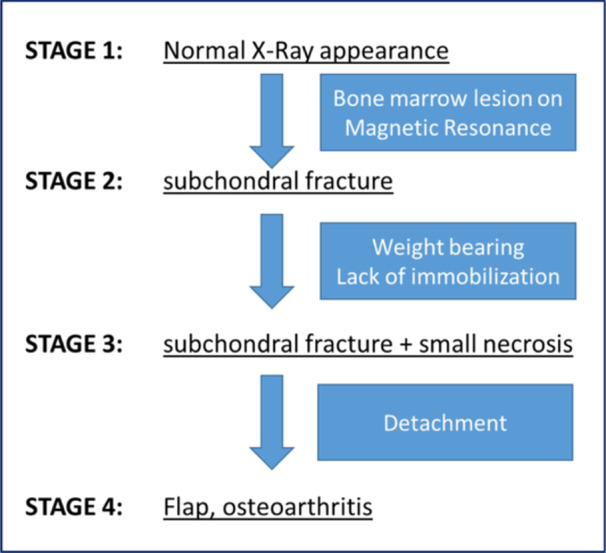
Pathophysiology of spontaneous osteonecrosis of the knee. In Stage 1, the radiographic appearance is normal. In Stage 2, only a subchondral fracture is recognized and there is no evidence of primary osteonecrosis. If patients continue weight bearing with lack of immobilization, the fracture does not heal and eventually the distal portion of the fracture may undergo flap formation with associated osteonecrosis. This is Stage 3. If weight‐bearing continues, the lesion undergoes detachment and finally results in the development of osteoarthritis. This is Stage 4.

**Figure 4:**
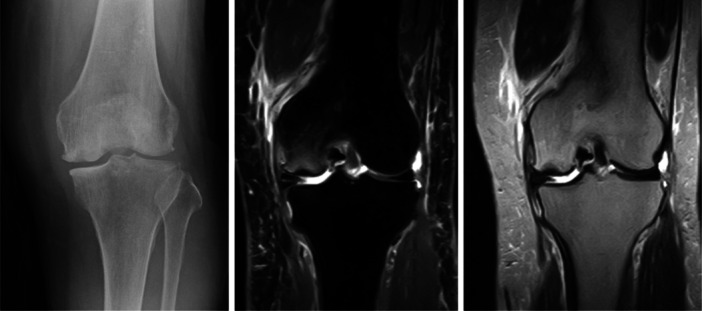
Stage 4 SIFK‐related osteonecrosis (or SONK) on plain radiographs and magnetic resonance imaging in an 85‐year‐old female patient. SIFK, subchondral insufficiency fracture. SONK, spontaneous osteonecrosis of the knee.

The concept of SIFK was later introduced based on a retrospective histopathological study of cases previously diagnosed as AVN in the femoral head [76] (Figures 1 and 2). This disease is generally seen in the shallow portion of the femoral head in elder women. At the onset of pain, radiographs show no apparent changes. Histologically, prominent fracture callus formation with associated granulation tissue is observed in the subchondral lesion, without any evidence of antecedent AVN [78]. SIFK is now considered a completely different disease from AVN. Conversely, a comparison between the clinical characteristics of SONK and SIFK suggested that they were almost similar, except for the typical location of each disease. A histopathological retrospective study using operative cases who received a clinical diagnosis of SONK was performed in 2000 [77]. In this paper, in radiologic Stage 2 (early stage) [32], only a subchondral fracture without any evidence of antecedent osteonecrosis was noted. In Stage 3, the presence of fracture, as well as tiny foci of fracture‐induced bone debris (osteonecrosis), was observed, despite the lack of an obvious AVN, indicating that the osteonecrosis seen in SONK was the result of subchondral fracture (Figures 3 and 4). It is important to keep in mind that following any fracture, necrosis of the immediately adjacent bone and bone marrow tissue is inevitable [54, 71]. Based upon this histopathological evidence, SIFK has been proposed as the true aetiology of SONK. Now, this controversy SONK vs SIFK seems to be a settled issue, with the original term of SONK interpreted as a misnomer, which therefore has been renamed as SIFK‐related osteonecrosis. This concept has been followed by several papers in the literature [27, 79].

A predictive model for SIFK classification has been proposed based on demographic and radiographic characteristics [52], with lateral meniscus extrusion or root tears, high‐grade lesions, SIFK located on the medial femoral condyle, and medial meniscus extrusion representing the most important factors in predicting progression to arthroplasty. These findings were confirmed by subsequent papers, with larger lesion sizes (anteroposterior/transverse), and articular surface collapse being highlighted as additional high‐grade markers [65]. More recently, Compagnoni et al. developed a six‐letter (A–F) topographic classification of BME in coronal knee MRI, which showed good reproducibility, providing an easy tool to describe the location and the size of BMLs [18]. The six‐letter system proved to be efficient in detecting very typical injury‐related BML patterns, like TLD (tibia‐lateral‐articular distal lesion) for ACL rupture and type E (Edge) for meniscal extrusion [19].

SIFK and AVN need to be clearly differentiated, since their true pathophysiology is completely different. The primary condition seen in AVN is a necrosis (infarction), with BML as well as subchondral fractures (collapse) developing only as secondary phenomena. On the other hand, the primary condition that leads to the development of SIFK is a fracture without any evidence of antecedent osteonecrosis. In considering the natural history and outcomes of SIFK and SIFK‐related osteonecrosis, several papers described the importance of the lesion size and width, with the prognosis considered to be benign if the lesion is small (<2 cm^2^) [37, 61]. Early diagnosis of SIFK may possibly affect the treatment as well as the prognosis of this condition.

## THE EFFECT OF SUBCHONDRAL BONE LOAD DISTRIBUTION ON BONE MARROW LESION DEVELOPMENT

Meniscus insufficiency may lead to OA, primarily due to the changes in the magnitude and pattern of stress distribution in the knee. Partial (16%–34%) meniscectomy has been shown to result in a >350% increase in contact force on the articular cartilage [66]. A study performed by Bedi et al. [8] measured pressure distribution under the medial meniscus in four different conditions: intact, post radial tears (30%, 60% and 90%), post meniscus repair and post partial meniscectomy. It was reported that focal stresses are created and increased as a function of radial tear, repair, and partial meniscectomy. More recent studies, however, documented that suture repair or resection of the superior leaflet are the best ways to maintain the force relationship in the knee joint [16], showing encouraging improvements even in the more complex root tears [14, 42]. On the other side, there is still no consensus on the impact of ramp lesions in load distribution and decision‐making [41], especially in combined meniscal‐ACL injuries [6, 34, 48].

Load/stress distribution may affect the cartilage surface, as well as the subchondral bone, eventually leading to BML, bone cysts, and pain. Restoration or replacement of the dysfunctional meniscus may help prevent these conditions. In this perspective, some evidence has been proposed with the use of a synthetic, non‐anchored, interpositional knee implant (NUsurface®, Active Implants) developed for patients with symptomatic meniscus insufficiency [23]. In a biomechanical study on human cadaveric knees, a comparison of pressure distributions measured under three conditions was reported, comparing intact natural meniscus, meniscectomized meniscus, and the resulting pressure under the synthetic, non‐anchored, interpositional knee implant [35]. The study showed that pressure distribution is affected by the condition of the medial meniscus, which could explain pain relief following treatment.

**Figure 5:**
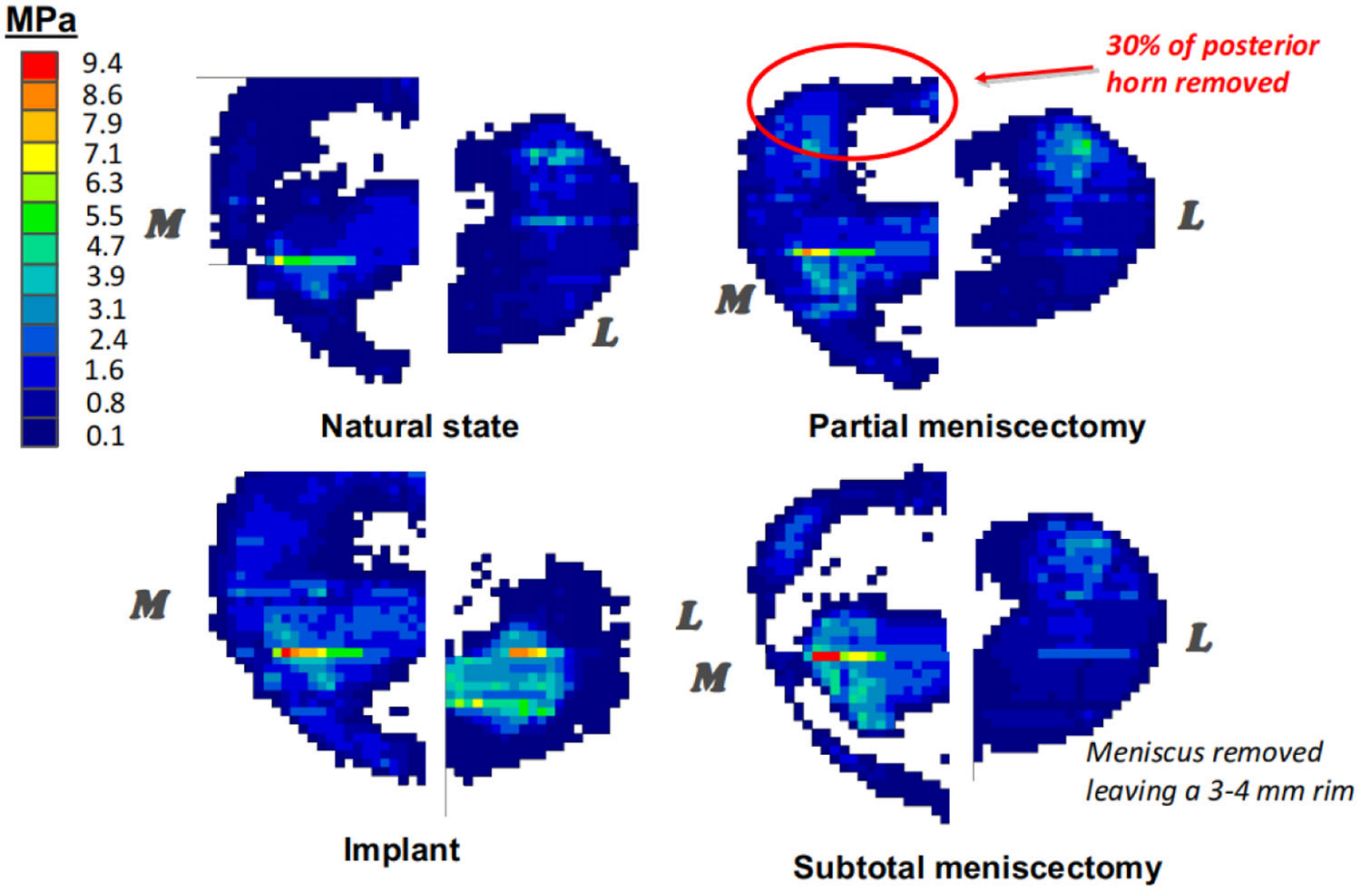
Medial (M) and lateral (L) pressure distribution measurements under an intact menisci (natural state), post partial meniscectomy, post sub‐total meniscectomy and post‐treatment with non‐anchored, interpositional knee implant.

It was also hypothesized that increased joint space and altered load sharing between both compartments is an important predicting factor. To explore this mechanism, the study was extended to measure the pressure distribution on the tibial surface of both medial and lateral compartments. Cadaveric knees were loaded under four conditions: (i) in the intact state, (ii) after a partial meniscectomy of the medial meniscus, (iii) after a subtotal meniscectomy of the medial meniscus, and (iv) after insertion of the non‐anchored, interpositional knee implant made of a synthetic, pliable material (Polycarbonate‐Urethane) reinforced with ultra‐high molecular weight polyethylene‐based fibres [67]. On the medial side, it was found that the non‐anchored, interpositional knee implant restored the contact area and reduced cartilage loads (near normal), compared to the meniscectomized knees (Figure 5).

**Figure 6:**
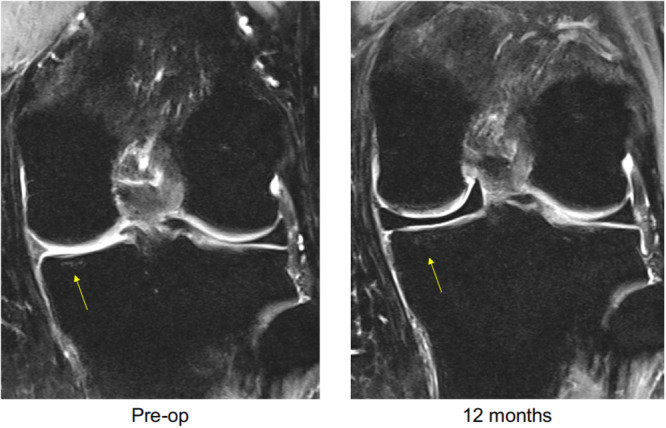
An example of a 51‐year‐old male who was diagnosed pre‐operatively as having a subchondral bone marrow lesion (left). Magnetic resonance imaging findings 12‐month post‐treatment with non‐anchored, interpositional knee implant (right).

On the lateral side, there was a slight decrease in the contact area and in both average and peak stresses compared to the intact and meniscectomized states. This may be explained by the shift in loading from the lateral to the medial side, compared to the meniscectomized knee. The changes observed in joint space between the medial and lateral compartments could be explained by this shifting loading pattern as well. While the insertion of the interpositional knee implant restored joint space on the medial side from 4 to 7 mm, there was no significant change in the lateral joint space. Clinical confirmation of the reported biomechanical findings was based on retrospective comparative MRI analyses conducted by a musculoskeletal radiologist, blinded to the subject's clinical data. Severity of BML in the medial femoral condyle and medial tibial plateau was evaluated at 1.5‐, 12‐ and 24‐month post‐treatment in 73 middle‐aged patients treated with this interpositional knee implant. The results showed a trend of improvement in BML between the follow‐up visits (12 and 24 months) and baseline (1.5 months) among patients with subchondral BML that was diagnosed using pre‐operative MRI scans. Specifically, 58% of patients presented with an improvement in BML of the medial femoral condyle and 25% showed improvement in BML on the medial tibial plateau, after 24 months. Figure 6 shows an example of a 51‐year‐old male patient that received the synthetic interpositional knee implant, 8‐month post‐meniscectomy. Pre‐operative and 12‐month post‐operative MRIs show improvement in the subchondral condition after implantation.

The results of these studies indicate that the meniscus plays a key role in preventing or developing subchondral BML, knee pain, and cartilage damage. Furthermore, it was demonstrated that the use of a synthetic, non‐anchored, interpositional knee implant might restore altered contact force and load distribution to the MFC in a meniscectomized knee to a more balanced, biomechanical condition. The medial tibial plateau, however, appears to be a more challenging compartment to improve. A recent review confirmed the benefits of meniscal implants [29], although highlighting the need for more studies to properly investigate and compare healing potentials and failure rates.

## SYSTEMIC FACTORS: THE RELATIONSHIP BETWEEN SUBCHONDRAL BONE ABNORMALITIES AND COAGULOPATHIES

Several hypotheses have been proposed for the pathogenesis of BMLs and osteonecrosis as stated above. Among these, disruption of the blood supply or venous occlusion has been claimed to result in increased intraosseous pressure [38, 69]. Besides, vascular disturbances with a mismatch between the arterial inflow and venous outflow or reduced vessel density may play a role. Accordingly, histological sections of non‐traumatic osteonecrosis frequently reveal thrombosis of terminal arteries within the subchondral bone [5]. For BML, obstruction of arteriolar inflow or venous outflow, injury to the vessel wall secondary to vasculitis, or altered lipid metabolism have been suggested as aetiologic factors [3, 55].

In relation to the intrinsic nature of BML and osteonecrosis, several studies have indicated that coagulation abnormalities causing thrombophilia or hypofibrinolysis might also contribute to their emergence. A literature analysis on thrombophilic or hypofibrinolytic parameters involved in the etiopathology of osteonecrosis or BMLs identified 50 studies including 2664 patients [50]. Eleven studies were published before 2000 and 39 after 2000, proving the current relevance of this topic. Nonetheless, no Level I study was identified. The majority of the studies focused on idiopathic or secondary osteonecrosis (45 studies; 2412 patients). The hip joint was reported as the most commonly affected joint (43 studies), followed by the knee (11 studies) and shoulder joint (4 studies). Treatment strategies have only been reported in 20 out of 50 studies (40%). Thirteen risk factors were described for the incidence of secondary osteonecrosis, including corticosteroids, alcohol, trauma, sickle cell disease, disseminated intravascular coagulation, tobacco, thrombotic thrombocytopenic purpura, biphosphonates, autoimmune diseases (i.e., systemic lupus erythematosus), chemotherapy, acquired immune deficiency syndrome, malignancy and Caisson disease (decompression sickness).

**Figure 7:**
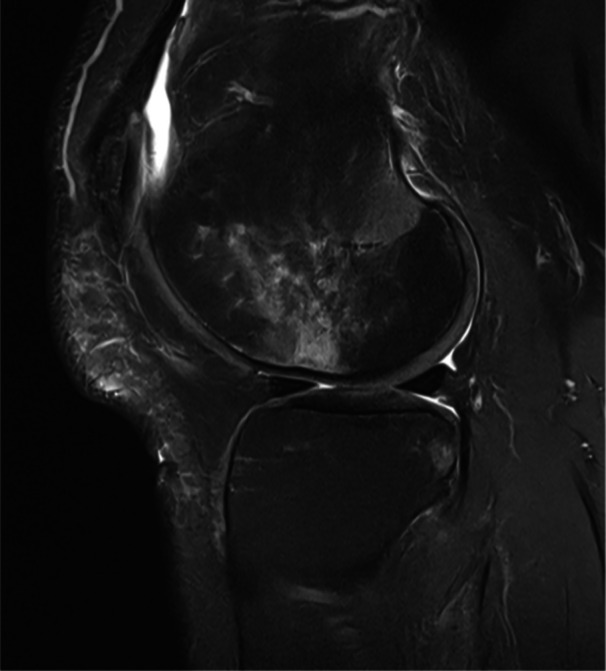
Magnetic resonance imaging (MRI—T2, sagittal) of a local bone marrow oedema at the right lateral femoral condyle of a 52‐year‐old patient with knee pain for 8 weeks and multilocular bone marrow oedema without trauma. Serological analyses revealed a disturbed lipid metabolism with an increase in lipoprotein (a). This patient may benefit from treatment with anticoagulants as a supplement to conservative physical therapy modalities.

Of utmost importance, a total of 53 thrombophilic or hypofibrinolytic parameters were specifically identified as possible risk factors for the emergence of osteonecrosis or BML [50]. The most frequently reported laboratory findings included altered serum concentrations of lipoprotein (a) (Figure 7), apolipoprotein A1, apolipoprotein B, decreased concentration and function of fibrinolytic agents (tissue plasminogen activator, protein C and protein S), and increased levels of thrombophilic markers (plasminogen activator inhibitor and coagulation Factor VIII). Furthermore, several single nucleotide polymorphisms (Factor V Leiden, methylene tetrahydrofolate reductase C677T, prothrombin 20210A, or tissue factor pathway inhibitor mutations) were identified in the molecular biological pathogenesis of osteonecrosis and BML. In the vast majority of studies, coagulation parameters were determined in the peripheral blood. Interestingly, Berger et al. [9] compared the serum laboratory findings with those locally determined in the affected bone marrow and demonstrated an elevation of parameters obtained from the bone analysis.

Against this background of coagulopathies playing a major role in the onset and pathogenesis of BML, anticoagulation therapy applying various pharmaceutical agents (i.e., warfarin, phenprocoumon, enoxaparin and direct oral anticoagulants) has been suggested for the drug treatment of this pathology. The prostacyclin analogue iloprost induces vasodilation and thereby influences the haemorheological properties in the end vascular bed. It simultaneously reduces the permeability of capillaries and inhibits the aggregation of thrombocytes and is approved for the treatment of pulmonary arterial hypertension and thromboangiitis obliterans. Thus, this drug treatment is also based on the vascular explanatory model for BML and osteonecrosis and may be considered particularly in patients with elevated lipoprotein (a) serum values [4].

**Figure 8:**
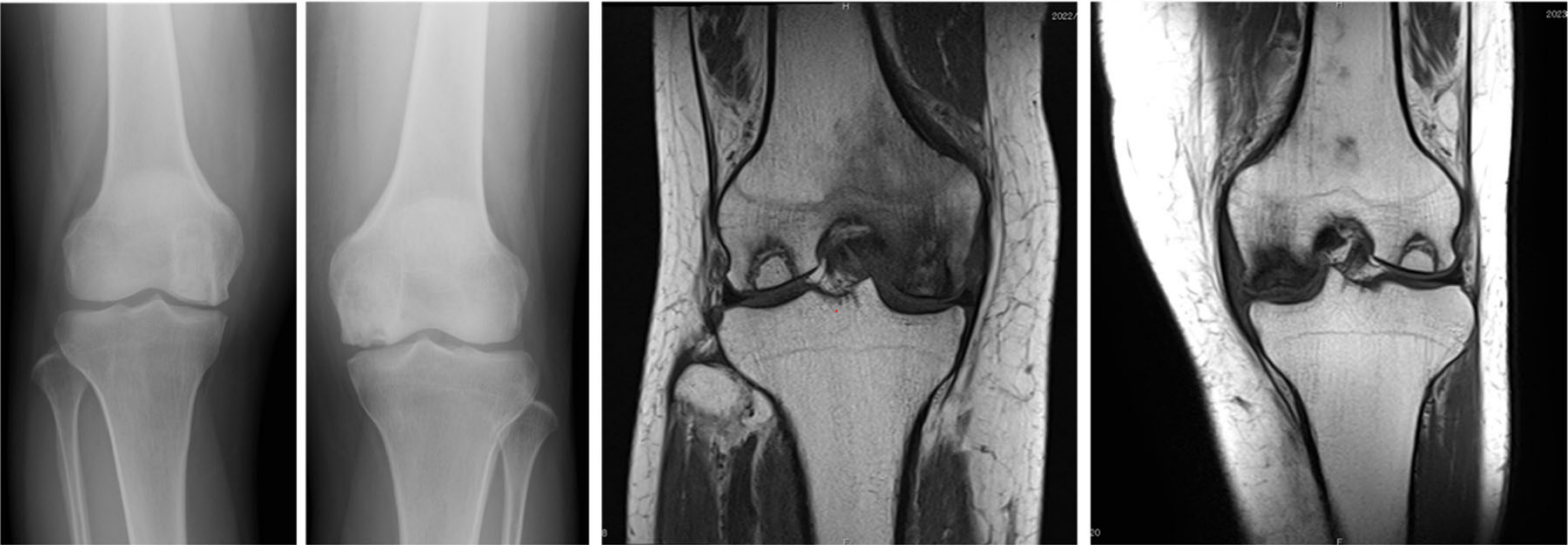
Plain radiographs and magnetic resonance imaging of corticosteroid‐associated bilateral knee osteonecrosis in a 42‐year‐old female patient.

In conclusion, the current evidence points to a broad variety of thrombophilic and hypofibrinolytic parameters contributing to the emergence of BML and osteonecrosis. Despite the heterogeneity in the reported results, the available data strongly suggest that coagulative disorders may play a key role in the pathogenesis of these subchondral bone abnormalities. Thus, determining lipoprotein concentrations and coagulation markers should be considered especially in patients with primary BML and osteonecrosis before prolonged corticosteroid use or chemotherapy [4] (Figure 8). These patients may benefit from treatment with anticoagulants as a supplement to conservative physical therapy modalities or surgical core decompression and total joint replacement.

## THE RELATIONSHIP BETWEEN HORMONAL FACTORS AND SUBCHONDRAL BONE

Subchondral bone and articular cartilage represent a functional unit. It is known that hormones, cytokines, and growth factors travel from the subchondral compartment to the articular cartilage and back from the overlying articular cartilage to the subchondral bone. It is important to take into account what the functional unit is exposed to during healthy states and also during the normal ageing process, and it must be taken into consideration that subchondral bone is also significantly affected by hormonal dysregulation. When studying subchondral bone diseases, such as traumatic osteochondral defects, osteochondritis dissecans, osteonecrosis and OA, it must be considered what is happening to the overlying articular cartilage. In the subchondral plate, there are hollow spaces, which provide a direct connection between the cartilage and the marrow cavity of the spongiosa, carrying nutrients and hormones to the basal cartilage. When these channels are damaged, the articular cartilage is dependent upon the synovial fluid as its sole source of nutrition.

Hormone effects act on two levels, systemic and locally within the joint environment. The hormones which affect the functional unit systemically include the calcium‐regulating hormones, parathyroid hormone (PTH), calcitriol (active Vit D) and calcitonin, sex hormones, oestrogen, testosterone and other hormones such as human growth hormone (GH), insulin‐like growth factor 1 (IGF‐1), thyroid hormones (T3 and T4), and cortisol [22, 51, 58, 70]. On a local level, cross‐talk between articular cartilage and subchondral bone is mediated by biological factors within the synovial fluid including B cells and T cells‐chemokines and cytokines, such as interleukin 1 (IL‐1), matrix metalloproteinases (MMPs), vascular endothelial growth factor (VEGF) and many others [53, 75]. Wingless/WNT [20] and TGF‐beta/BMP [11] signalling pathways were found to play important regulatory roles as well. These are complex pathways regulated by various secreted agonists and antagonists, which provide tight regulation to maintain normal joint homoeostasis.

GH affects several tissues, including the liver, muscle, kidney and also subchondral bone. The general systemic effects of GH and IGF‐1 are well‐known and documented [22]. However, the exact role of GH/IGF‐1 in maintaining normal subchondral bone and cartilage in the adult is less clear. IGF‐1 plays an important anabolic role in cartilage/subchondral bone and also presents anti‐catabolic activity [22]. IGF‐1 can be synthesized locally by chondrocytes in both an autocrine and paracrine manner [33] and it has been shown to stimulate matrix production by cartilage cells [60]. IL‐1 inhibits cartilage matrix production, stimulates catabolic activity in cartilage, and plays a role in the development of OA [49]. IGF‐1 can block the ability of IL‐1 to stimulate proteoglycan degradation and can restore proteoglycan synthesis. There is also evidence that IGF‐1 is beneficial to injured cartilage [22]. Inhibiting the activity of IL‐1 and/or increasing the activity of IGF‐1 in cartilage could therefore represent a potential benefit in preventing cartilage degradation and progression to OA. IGF‐1 and GH decrease with age and an animal model of OA also showed an age‐related decline in the responsiveness of chondrocytes to the anabolic effects of IGF‐1 [36], with OA severity increasing significantly with age, which suggests that a lack of responsiveness to IGF‐1 may play a role in the pathogenesis of this disease [22].

Sex hormones are important regulators of cartilage and subchondral bone biology. Sexual steroids, oestrogen, progestogens and androgens influence cartilage quality and bone mass. Oestrogen regulates cartilage, muscle synovium, ligaments and subchondral bone by increasing proteoglycan production in chondrocytes, regulating intracellular calcium concentration in chondrocytes, and it has been shown to decrease cartilage damage in several animal models [15, 68]. Oestrogens prevent OA development with a direct action on chondrocytes and [17] beta‐estradiol reduces the expression of MMPs associated with cartilage matrix degradation in OA. Oestrogens influence joint tissue metabolism at many crucial levels and through several complex molecular mechanisms. All these effects of oestrogens on the joints are either significantly dampened or even lost following menopause. Less is known about the direct effects of testosterone on cartilage. However, chondrocytes from both male and female donors express receptors for testosterone and DHT. Androgens penetrate the chondrocytes, where they are transformed into oestrogens and DHT. Several studies concluded that androgens protect against cartilage degradation [70].

Thyroid hormones are also very important for the functional unit. T3 is a positive regulator of cartilage maturation, stimulating the clonal expansion of resting chondrocytes and their subsequent hypertrophic differentiation [51], and is significantly involved in the cross‐talk signalling pathways. T3 and T4 increase alkaline phosphatase and type X collagen production [59], and the presence of T3 receptors on articular cartilage chondrocytes explains their T3‐induced terminal differentiation, which has been suggested to have a pathogenic role in the development of OA and crystal deposition diseases [56]. Thyroid hormones also play a crucial role during development, linear growth, and adult bone turnover and maintenance. In‐vivo studies demonstrated anabolic actions of T3 during growth and catabolic effects on adult bone. Thus, an excess of thyroid hormones, as in course of thyrotoxicosis, has been proved to cause secondary osteoporosis [7].

The relationship between subchondral bone, hormones and nutrients occurs from both sides of the functional unit, from the subchondral bone upwards and from the articular cartilage downwards. To treat joint diseases, this essential knowledge is paramount to designing appropriate treatment strategies to maintain healthy communication within the functional unit. Further research should investigate the role of hormones in the case of BML and osteochondral injuries, from a prognostic but even therapeutic point of view. Finally, considering the large use of injective therapies in the treatment of chondral injuries of the knee and more recently in the delivery of substances at the subchondral bone level [31], the exact makeup of the catabolic and anabolic components injected should be studied in relation to systemic and local hormonal factors and mediators, to understand how they may affect the various pathways within the cartilage and subchondral bone unit.

## CONCLUSION

BMLs of the knee are a common MRI finding. These alterations of the bone marrow signal intensity are present in a wide range of pathologies, traumatic and non‐traumatic, and may be expressions of reversible or irreversible conditions. While the field is still heterogeneous in the definition of the nosographic framework, there is a trend with the convergence towards a common terminology, which could help to shed more light on the complex and variegate field of BML. This narrative review clarified BML features, development, and classification, aiming at providing clinicians with clear indications for the correct diagnostic process and treatment strategies for these common yet still largely unknown lesions.

Future studies should focus on better understanding the etiopathogenetic mechanisms, including the complex interaction of mechanical factors like local load distribution and the local or systemic biological factors, which can concur to the development of BML from one side, and, on the other hand, may represent targets for future treatments to address BMLs and preserve or restore the osteochondral unit.

## AUTHOR CONTRIBUTIONS

*Conceptualization*: Filardo Giuseppe, Andriolo Luca and Shabshin Nogah. *Writing—original draft preparation*: Sangiorgio Alessandro, Galea Anthony, Shabshin Nogah, Linder‐Ganz Eran, Yamamoto Takuaki and Orth Patrick. *Writing—review and editing*: Andriolo Luca and Filardo Giuseppe. *Supervision*: Filardo Giuseppe. All authors have read and agreed to the published version of the manuscript.

## CONFLICT OF INTEREST STATEMENT

ELG is affiliated to Active Implants, Netanya, Israel. The other authors declare no conflict of interest.

## ETHICS STATEMENT

No ethical committee approval or patient consent was needed due to the nature of the study.
